# The Eleventh Annual Announcement of College N. Y. Ophthalmic Hospital

**Published:** 1889-07

**Authors:** 


THE ELEVENTH ANNUAL ANNOUNCEMENT OF THE COLLEGE OF THE NEW YORK OPHTHALMIC HOSPITAL, for the Session of 1889 and 1890, is received.

This college teaches the treatment of eye diseases upon homœopathic principles. The State of New York has granted it the right to confer the degree: *Oculi et Auris Chirurgus*. The professors are: Henry C. Houghton, M. D., Aural Pathology and Therapeutics; Sympathetic Ophthalmia; George S. Norton, M. D., Diseases of the Retina and Optic Nerve; Wm. E. Rounds, M. D., Diseases of the Middle Ear and of Lids and Lachrymal Apparatus; F. H. Boynton, M. D., Diseases of the Bulbus and Orbit; Affections of the Muscles; Chas. Deady, M. D., Diseases of the Lens and Humors of the Eye; Glaucoma; N. L. McBride, M. D., Refraction and Accommodation of the Eye; Charles C. Boyle, M. D., Diseases of Uveal Tract; Relation of Diseases of the Eye to General Diseases; A. B. Norton, M. D., Diseases of the Conjunctiva, Cornea, and Sclera. From the above list of professorships it will be seen that a very thorough instruction is given in the important subject of Ophthalmology.

